# Comparison of the diagnostic concordance of tele-EMS and EMS physicians in the emergency medical service—a subanalysis of the TEMS-trial

**DOI:** 10.3389/fdgth.2025.1519619

**Published:** 2025-04-30

**Authors:** Patrick P. Hess, Michael Czaplik, Johanna Hess, Hanna Schröder, Stefan K. Beckers, Andreas Follmann, Mark Pitsch, Marc Felzen

**Affiliations:** ^1^ Department of Anesthesiology, Medical Faculty, RWTH Aachen University, University Hospital RWTH Aachen, Aachen, Germany; ^2^ Department of Hematology, Oncology, Hemostaseology, and Stem Cell Transplantation, Faculty of Medicine, RWTH Aachen University, Aachen, Germany; ^3^ Docs in Clouds TeleCare GmbH, Aachen, Germany; ^4^ Aachen Institute for Rescue Management & Public Safety, City of Aachen and University Hospital RWTH Aachen, Aachen, Germany

**Keywords:** telemedicine, EMS-physician, paramedics, diagnostic concordance, TEMS-study

## Abstract

**Introduction:**

The emergency medical services (EMS) in Germany are facing several challenges in the near future. Due to the increasing number of emergency missions, the availability of EMS physicians is becoming more limited, resulting in longer response times. To maintain the high quality of EMS, telemedical support systems have shown potential as a valuable complement to the existing system for specific diagnoses. Since 2014, a tele-EMS system has been implemented in Aachen as an integrated telemedical solution alongside standard EMS. Accurate prehospital diagnosis plays a crucial role in ensuring appropriate hospital admission and reducing the time to clinical treatment for time-sensitive conditions. The main TEMS study demonstrated the overall non-inferiority of tele-EMS physicians compared to on-site EMS physicians. This sub-analysis focuses on comparing the diagnostic accuracy between these two groups.

**Methods:**

Up to four prehospital diagnoses were selected, coded according to the ICD-10 system, and compared with all admission and discharge diagnoses.

**Results:**

The comparison between diagnoses made by tele-EMS physicians and on-site EMS physicians with admission diagnoses showed no significant difference (*p* = 0.877). Additionally, no significant differences were found for the diagnoses of stroke (*p* = 0.385) and epileptic seizure (*p* = 0.738). However, patients from missions where paramedics decided to consult a tele-EMS physician had significantly longer hospital stays compared to those from missions where an on-site EMS physician was initially dispatched (*p* < 0.001).

**Discussion:**

This randomized controlled analysis demonstrated that there is no difference in diagnostic accuracy between on-site EMS physicians and remote tele-EMS physicians. The significantly longer hospital stays for patients treated by tele-EMS physicians suggest that EMS physicians may be called too frequently for non-severe cases.

**Clinical Trial Registration:**

clinicaltrials.gov, identifier (NCT02617875).

## Introduction

Due to the increasing number of emergency missions the availability of physicians is becoming more limited, leading to longer response times (1, 2). Another factor is the challenge of staffing all shifts with EMS physicians, especially in rural regions where availability is difficult to ensure (3). The high number of non-severe missions could be a starting point for innovative technology and support systems to assist paramedics on scene with a remote EMS physician (4, 5). Additionally, telemedical support has shown to provide benefits even in life-threatening missions or to assist on-site EMS physicians in complex cases (6).

In Germany, approximately 56% of all emergencies are managed independently by paramedics, who can request an EMS physician if necessary (1). In the remaining 44% of cases, where an EMS physician is involved, paramedics are typically the first to arrive on-site, conducting the initial assessment and treatment (7). The delayed arrival of EMS physicians is caused by a higher concentration of paramedic stations compared to EMS physician locations (3).

In 2007 a pilot program was launched in the City of Aachen to evaluate the potential use of telemedical support in EMS (8). After an initial testing phase, the telemedical support system demonstrated its benefits for EMS (9, 10) and the technical requirements were determined (11). Since 2014 the telemedical support system has been fully implemented in Aachen as a holistic telemedicine system, operating 24 h a day (12). One of its key advantages is reducing the need for on-site EMS physicians without compromising quality of care (7, 13, 14). This benefit is particularly beneficial in rural areas (15–17). Additionally, tele-EMS consultations can help bridge the gap until an EMS physician arrives on scene (18). The administration of medication by paramedics has also been shown to be feasible with the guidance of a tele-EMS physician (19–21).

There is a lack of recent data on the diagnostic accuracy of telemedical emergency care, and only a few studies have investigated this aspect. Previous research have shown that the accuracy of prehospital diagnoses has improved significantly over recent decades, reaching a relatively high level for EMS physicians (22–24). Accurate diagnoses are crucial ensuring appropriate hospital admissions (23) and minimizing the time to treatment initiation for time-critical conditions such as cerebrovascular ischemia or ischemic heart disease (25–27). A retrospective study, analyzing tele-EMS physician mission data in Aachen found no significant differences in diagnostic concordance, except for epileptic seizures. In these cases, both on-site and tele-EMS physicians more frequently misdiagnosed seizures as a stroke, likely reflecting a cautious approach to a critical condition (28).

As the telemedical support system was a new approach to prehospital care in EMS, the TEMS study (29, 30) investigated the feasibility of general implementation for specific diagnoses under randomized and controlled conditions. The main findings demonstrated the non-inferiority regarding patient outcomes and adverse events, as well as improved documentation quality, and medical history collection (30).

This sub analysis of the TEMS RCT investigates the non-inferiority of tele-EMS physicians in diagnostic accuracy compared to on-site EMS physician. Additionally, it aims to determine if the data from the retrospective study can be confirmed under prospective randomized controlled conditions.

## Methods

### Study design

The prospective, randomized interventional, controlled, open-label, two-arm, parallel-group sequential single center TEMS trial investigated the non-inferior application of a telemedical support system in the urban EMS of the city of Aachen, Germany. To ensure the statistical significance of the data the sample size was determined to be 3,010 patients through a statistical power analysis. Randomization was performed by dispatching software, considering previously defined symptom complexes and emergency keywords assessed by staff at the dispatch center during emergency calls. The detailed description, study protocol and trial were published previously by Stevanovic et al. (29, 30).

After receiving the emergency call and determining that a physician was required, the randomization software assigned missions into two groups: treatment by a tele-EMS physician or treatment by an EMS physician on scene.

While the EMS physician was often present before the paramedics had completed their assessments and diagnostics, the tele-EMS physician was only alerted to join the mission if deemed necessary by the paramedics. This study specifically focused on comparing the quality of prehospital diagnoses, with the primary outcome parameter being the concordance between prehospital and clinical diagnoses. Secondary outcome parameters included the overall length of hospitalization and the duration of stay in an intensive care unit (ICU).

### EMS in Germany

Germany operates a two-tier system of EMS, consisting of either an ambulance with two paramedics or a combination of an ambulance with a physician-staffed emergency vehicle, where an EMS physician works along with another paramedic. Both teams are located in separate vehicles and can meet at the scene if necessary (called “Rendezvous” system) (31).

Previously, emergency care provided by paramedics was limited by legal restrictions, which could prevent them from providing the best possible therapy in a timely manner or lead to conflicts within the paramedic team (32). Additionally, paramedics often had to request an EMS physician to delegate the administration of medications due to legal constraints (12). Before 2014, only licensed physicians were allowed to administer medications or delegate their administration (33). Since a legal reform in 2014 paramedics are now required to undergo a three-year education program to acquire professional knowledge of prehospital emergency medicine. This training allows for certain tasks previously reserved for physicians to be delegated to paramedics (34, 35).

### Data source

All patients treated by prehospital physicians who received a documented admission diagnosis from the emergency department (ED) physician or discharge diagnosis from the clinical physician were included in the statistical analysis. Only patient datasets with valid diagnoses were considered, datasets containing incomplete or inadequate data were excluded. Prehospital diagnoses were documented either by hand or by selecting a tracer diagnosis on a standardized emergency protocol. The tele-EMS physician used a digital form with the same relevant information.

Additionally, the length of hospital and ICU stay were recorded for all patients. The durations of stay were compared between missions with concordance of the diagnosis from the tele-EMS physician and the EMS physician and missions without a match in those. For ICU-stays, only patients with a recorded duration of stay of at least one day were included in the analysis.

### Diagnose matching

For the statistical analysis, the prehospital diagnoses had to be converted to the International Statistical Classification of Diseases and Related Health Problems-10-German Modification (ICD-10-GM) code. The German Modification (GM) is the adapted version of the ICD-10-WHO for the German outpatient health care system.

The prehospital diagnoses were assigned to the corresponding main group of the ICD-10-WHO to ensure comparability. All admission and discharge diagnoses from the hospital were documented as an ICD-10-GM code and retrieved from the hospital documentation system.

The diagnostic accuracy was analyzed by comparing the prehospital diagnoses of all admission and discharge diagnoses of the hospital.

For prehospital diagnosis up to four documented diagnoses were considered. A match was considered if one of the four prehospital diagnoses was also found in the admission or discharge diagnoses. The ICD code was compared without the specific diagnosis number (e.g., I99.*), as there is no need to specify a diagnosis in the EMS.

### Statistical and documentation tools

The prehospital and hospital data were collected using the OpenClinica documentation tool, Version 3.14 (OpenClinica LLC, Waltham, Massachusetts, USA) and manually entered into an electronic case report form. The database was exported to Microsoft Excel, Version 2012 (Microsoft, Redmond, Washington, USA). Statistical analyses were performed using SPSS Statistics, Version 25 (IBM Corporation, Armonk, New York, USA) for Windows. For the statistical analysis, the Chi-square test was conducted with a degree of freedom = 1. The testing for normal distribution was carried out with the Shapiro–Wilk test. The analysis of the duration of hospitalization was done using the Wilcoxon-Mann–Whitney test. Significance was assumed to be at a level less than 5%.

### Ethics and dissemination

The statistical analysis plan for the trial was published before the database was closed (36). The Ethics Committee of the University of RWTH Aachen, Germany approved the study with reference number EK 170/50 on November 23, 2015. Changes to the study protocol were approved on September 20, 2016. The study is registered in the ClinicalTrials.gov registry under reference number NCT02617875 (29).

## Results

We compared the diagnostic concordance of at least 537 missions with treatment by tele-EMS physicians and 1,204 missions with treatment by EMS physician.

The gender of the patients was documented in 1,734 missions. 927 (53.7%) of the patients were female. The average age was 63 years with a standard deviation of 22 years.

**Figure 1:**
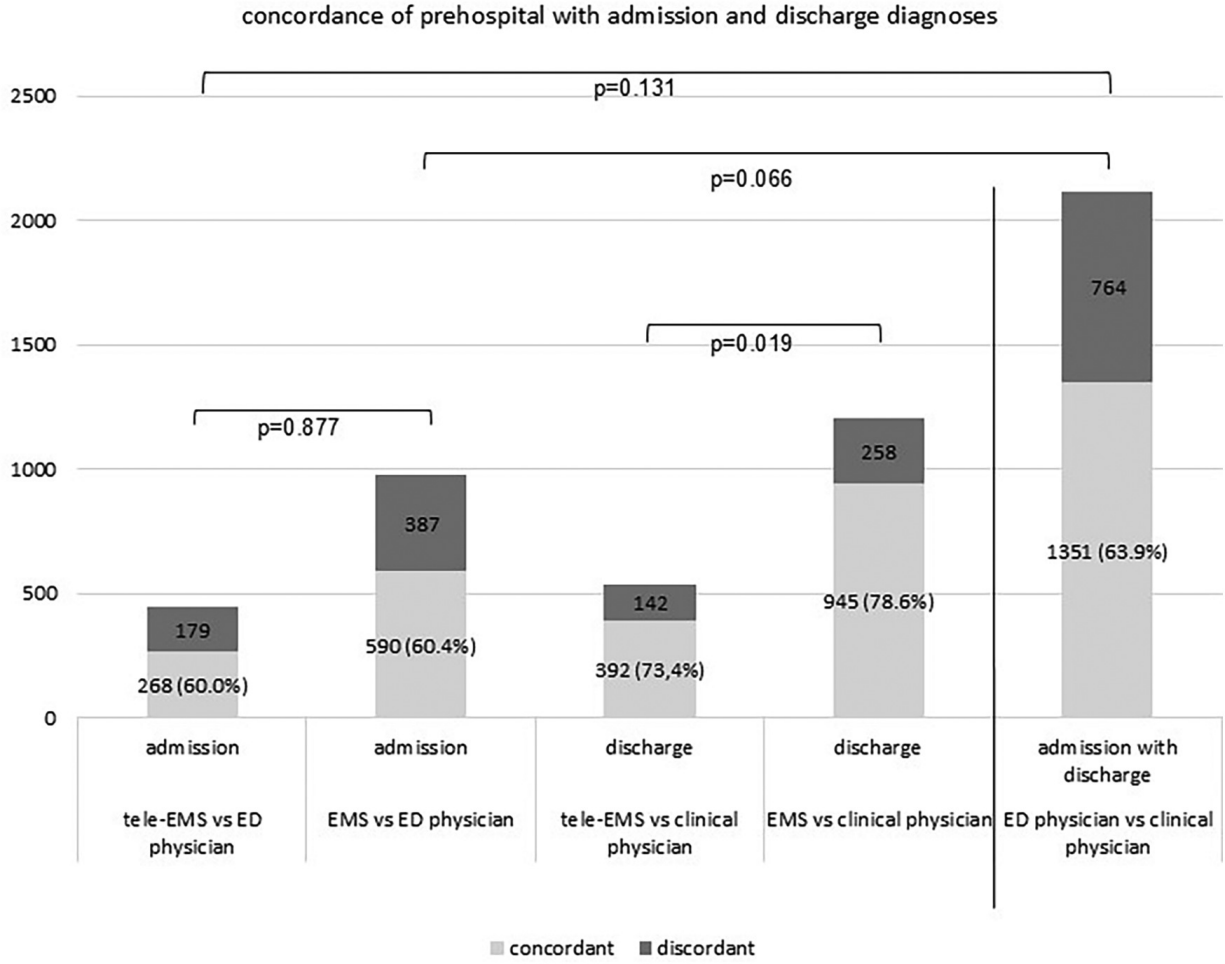
Demonstrates the diagnostic accordance of the prehospital selected diagnoses by tele-EMS and EMS physicians compared with admission diagnoses selected by ED physicians and discharge diagnoses selected by clinical physicians. The ratio of concordant cases to the total number is indicated in brackets.

Admission diagnoses were documented in 447 missions treated by tele-EMS physicians and 977 missions treated by EMS physicians. Regarding the concordance of the discharge diagnosis, we compared 534 missions treated by tele-EMS physicians and 1,203 missions treated by EMS-physicians (Figure 1).

**Figure 2:**
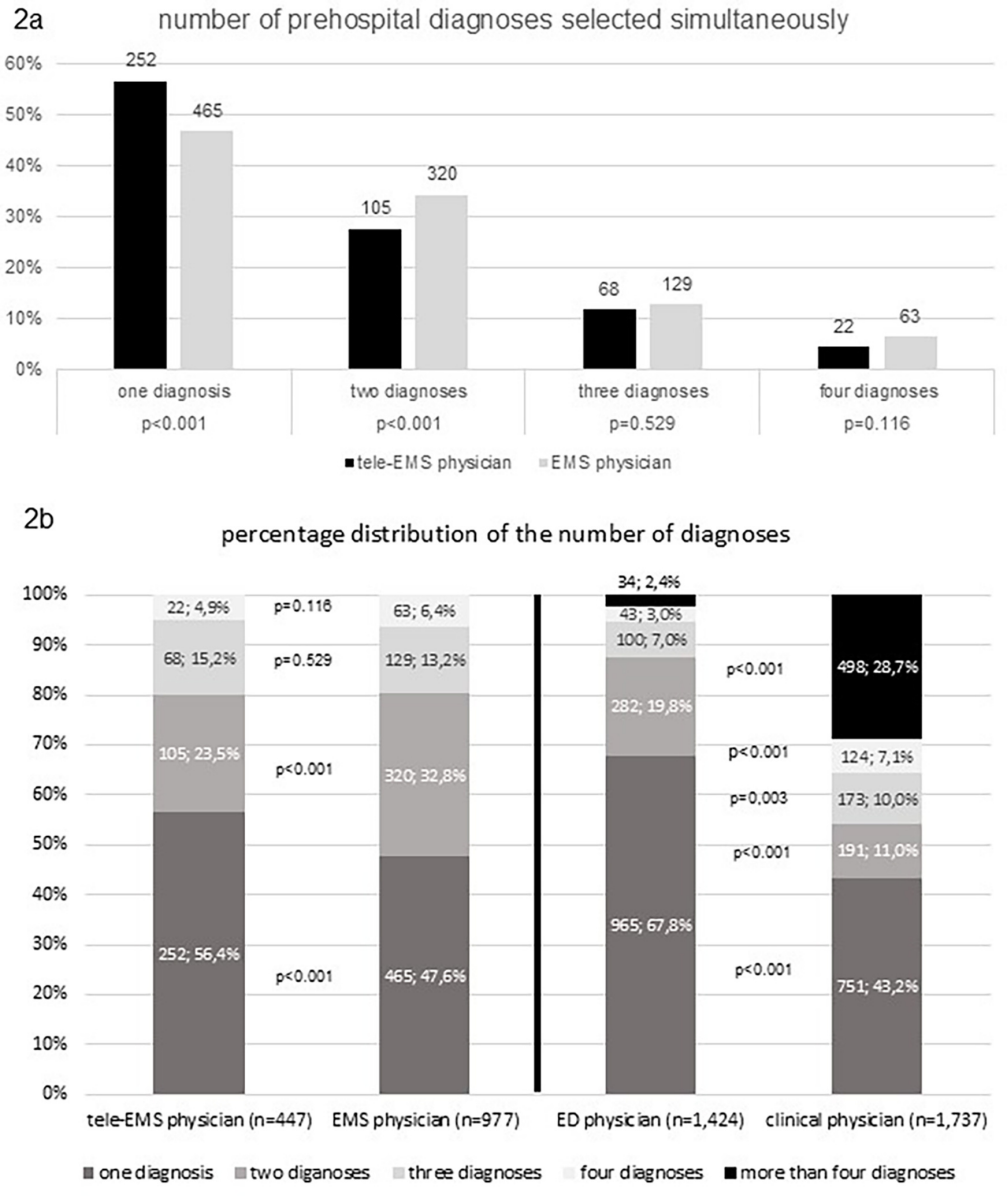
**(a)** shows the percentage comparison of the number of selected diagnoses between tele-EMS and EMS physicians; **(b)** shows the percentage comparison of the distribution of selected prehospital, admission and discharge diagnoses.

An admission diagnosis was significantly less frequently recorded than the discharge diagnosis (*p* < 0.001). There were no significant differences between tele-EMS physicians and EMS physicians regarding the admission diagnoses (*p* = 0.877). However, the number of cases in which at least one prehospital diagnosis matched the discharge diagnosis was significantly higher for EMS physicians than for tele-EMS physicians (*p* = 0.019). As depicted in Figure 2, EMS physicians recorded two diagnoses significantly more often than tele EMS physicians (*p* < 0.001). Additionally, there was a significant difference in the concordance between admission and discharge diagnoses for missions involving both tele-EMS physicians and EMS physicians. There is no significant difference in the concordance between admission diagnoses selected by the ED physician and discharge diagnoses selected by the clinical physician, compared to the concordance of prehospital diagnoses by the tele-EMS physician and EMS physician to the admission diagnoses.

As illustrated in Figure 2, a statistically significant higher frequency of hospital records containing more than four discharge diagnoses was observed (*p* < 0.001).

The analysis of the most common main diagnoses showed no significant difference between tele-EMS and EMS physicians. The results are displayed in Table 1. The diagnoses of stroke and epileptic seizure included in the diagnostic main group neurological emergencies (NE) were analyzed separately. For both diagnoses no significant differences were found (stroke: *p* = 0.385; seizure: *p* = 0.738).

**Table 1:** Displays a comparison between tele-EMS physicians and EMS physicians regarding the diagnostic concordance of medical main diagnoses.

Medical diagnosis main groups	Matches tele-EMS physician	Matches EMS physician	Chi-square test (p)
Acute coronary syndrome	72 (78.3%)	120 (78.9%)	0.899
Rhythm disorder	28 (62.2%)	53 (77.9%)	0.069
Cardiovascular disorder	56 (70.0%)	157 (74.4%)	0.449
Pulmonary disorder	21 (58.3%)	53 (69.7%)	0.234
Neurologic disorder	86 (72.9%)	132 (69.5%)	0.523
Stroke	62 (71.3%)	84 (65.6%)	0.385
Seizure	14 (77.8%)	31 (81.6%)	0.738
Intoxication	5 (62.0%)	21 (75.0%)	0.486
Psychiatric disorder	4 (57.1%)	15 (62.5%)	0.798
Metabolic disorder	4 (57.1%)	12 (92.3%)	0.061
Acute abdomen	16 (88.9%)	63 (92.6%)	0.604
Anaphylaxis	7 (87.5%)	14 (100%)	0.176
Abdominal disorder	37 (80.4%)	171 (84.7%)	0.483
Orthopaedic disorder	31 (72.1%)	78 (83.9%)	0.109
Trauma or Injuries	19 (90.5%)	65 (87.8%)	0.662
Suspected infection	4 (100%)	3 (100%)	–
Other	2 (50.0%)	7 (75.0%)	0.157

**Figure 3:**
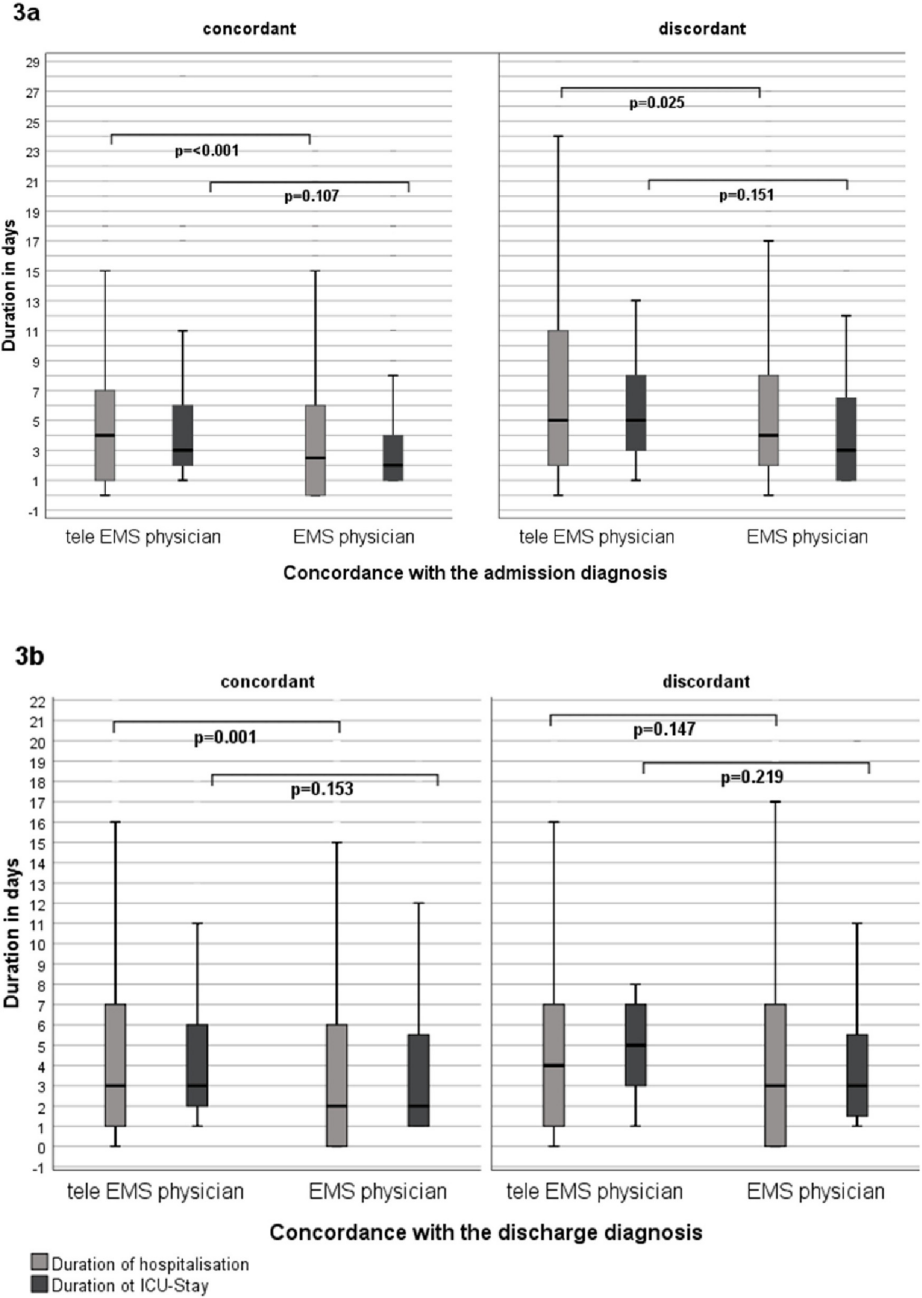
**(a)** shows the duration of the hospitalization and the ICU-stay between tele EMS physician and the EMS physician in comparison to the concordance with the admission **(b)** and the discharge diagnosis.

If the prehospital and admission diagnoses were concordant, the length of hospital stay was significantly longer in missions with treatment by the tele-EMS physician than in missions with treatment by the EMS physician (*p* < 0.001). Furthermore, missions with discordance between the diagnoses of the tele-EMS physician or EMS physician and the admission diagnoses also showed a significant difference (*p* = 0.025). However, the length of hospital stay was longer if the diagnoses of the tele-EMS physician or EMS physician differed from the hospital's diagnoses. Regarding ICU stay, there was no difference between the tele-EMS physician and the EMS physician in terms of concordance with the admission diagnoses (*p* = 0.107) or discordance (*p* = 0.151) (Figure 3).

## Discussion

This sub analysis of the prospective, randomized controlled TEMS study investigates the diagnostic concordance between tele-EMS physicians and EMS physicians.

Regarding the admission diagnosis, there was no significant difference between the prehospital diagnosis made by either the tele-EMS physician or the EMS physician. The results indicate that the prehospital diagnosis is not influenced by the presence of an EMS specialized physician on-site or in the ED. The admission diagnosis from the ED physician did not deviate more frequently from the prehospital diagnosis than the admission diagnosis itself did from the prehospital diagnosis. Both prehospital physicians reached similar conclusions by interpreting symptoms and patient history without additional diagnostic tools. However, it is important to note that the admission diagnosis could be influenced by a selection bias related to the prehospital diagnosis.

A key question is whether the tele-EMS physician is more limited in diagnostic resources than the emergency physician. The fact that the tele-EMS physician significantly more often selects only one diagnosis underlines the ability to make a remote decision based on a precise handover by the paramedics, combined with the transmitted vital signs. This demonstrates the feasibility of obtaining all critical medical information via telemedicine, enabling a focused approach using a single “Standard Operating Procedure” (SOP). In contrast, the EMS physician more frequently selects two diagnoses, and being present on scene can sometimes lead to a broader focus, potentially diluting attention to the essential aspects due to the availability of unfiltered information. On the other hand, a less comprehensive clinical picture of the patient's condition may result from the absence of tele-EMS. The tele-EMS physician's ability to provide a comprehensive diagnosis may be constrained due to the limitations inherent to remote consultations. The absence of the physician from the patient's immediate vicinity during clinical assessments may result in the omission of vital clinical observations that cannot be effectively captured by technological aids. Especially in relation to a physical examination of the patient, a physician who is physically present may have an advantage.

The overall concordance between prehospital diagnoses and discharge diagnoses was higher than with the admission diagnoses. This can be attributed to the hospital's practice of selecting more than four discharge diagnoses for completeness, the variety of diagnostic possibilities, and billing considerations. Similarly, the EMS physician more frequently selects two diagnoses than one, which is likely why the diagnoses made by EMS physicians aligned more frequently with the discharge diagnoses than those made by the tele-EMS physicians.

It is important to consider the lack of diagnostic tools outside of the hospital. Recent literature suggests a benefit in prehospital diagnostic accuracy using predictors or ultrasound as a diagnostic tool in specific conditions, especially in missions involving dyspnea, unclear circulatory issues, or abdominal pain (24, 37, 38). In the field of telemedicine, paramedics could perform an ultrasound examination and receive support in interpretation by a tele-EMS physician through digital transmission.

The retrospective analysis conducted by Quadflieg et al. showed that diagnostic concordance for specific diagnoses did not differ significantly between tele-EMS physicians and EMS physicians, except for the diagnosis of “epileptic seizure” (28). Our study demonstrated an improvement in the diagnostic accuracy of tele-EMS physicians under randomized controlled conditions, with a higher number of missions, showing no significant difference in the diagnosis of “epileptic seizure”. This finding could explain the higher diagnostic accuracy observed by Quadflieg et al. Retrospective analyses have previously suggested that tele-EMS and EMS physicians provide comparable diagnostic accuracy for certain diagnoses, such as acute coronary syndrome (28, 39).

The overall length of hospital stays was longer for patients treated by the tele-EMS physician. However, it is important to note that the tele-EMS physician was only involved in cases where paramedics deemed physician support necessary. In contrast, the EMS physician arrived on scene faster, before paramedics had completed their assessment and diagnostics to decide whether to call for a physician. This explains the higher number of missions handled by the EMS physician, indicating that the threshold for alerting EMS physicians in the city of Aachen may be too low, leading to an underutilization of paramedic skills.

This is further supported by the fact that there is no significant difference in the length of ICU stays between patients treated by tele-EMS physicians and those treated by EMS physicians. It can be assumed that ICU admissions are largely driven by the severity of the patient's condition, which cannot be prevented by either tele-EMS or EMS physicians.

Considering additional factors, there was a difference in the duration of hospitalization in missions with concordant as well as with discordant diagnoses. As shown in Figure 3, hospital stays were longer in cases with discordant diagnoses made by both tele-EMS and EMS physicians, likely due to the complexity of the diagnosis. Conversely, a significant difference was observed when the prehospital diagnosis was correct, which can be attributed to the fact that paramedics only consult the tele-EMS physician when necessary.

In the context of the existing literature, the use of telemedicine structures is a useful addition to the existing system of standard EMS. Quick and easy availability of tele-EMS support enhances patient safety by ensuring timely and accurate diagnoses, especially in situations where EMS physicians may have limited diagnostic resources. Additionally, tele-EMS provides backup for paramedics. Further research is necessary to achieve an equivalent study size. Additionally, future studies should consider the severity of emergency situations and examine the outcomes of different parameters.

## Limitations

The missions that did not involve the tele-EMS physician were not included in the study, leading to a lack of documented diagnoses for these cases. It can be assumed that no consultation occurred in clear cases where paramedics did not require physician support. In the other study group, the conventional EMS physician was automatically alerted and always on-site. If these were indeed clear cases for the paramedics, we could expect high diagnostic quality from the tele-EMS physician as well, which would strengthen the results. Unfortunately, there are no documented diagnoses for cases where paramedics did not consult a tele-EMS physician. To avoid this bias, future studies should include a clear consultation requirement.

Another limitation of the study is the assignment of freehand diagnoses and their conversion into the ICD system. In some cases, diagnoses were not assigned in a way that directly aligned with the categories of the ICD system, which required certain diagnoses to be mapped analogously. This process may have introduced potential bias, as the conversion of freehand diagnoses into standardized codes was not always precise or objective. Another potential influencing factor on the accuracy of the diagnoses is the practice of hospital coding. In some instances, coding was not solely driven by medical considerations but was also influenced by billing requirements, which could further introduce bias. These factors may have impacted the reliability of the diagnostic data and distorted the comparability of the results.

## Conclusion

The quick availability of tele-EMS support can improve patient safety by ensuring timely and accurate diagnoses, particularly in situations where EMS physicians may have limited diagnostic capabilities. As tele-EMS systems continue to evolve, they could provide a valuable backup in emergencies, allowing paramedics to focus on delivering high-quality care. The results from the Aachen study could be expanded to other regions and countries with different healthcare systems. Multicenter studies, including diverse geographic locations and patient populations, would help generalize the findings and determine if tele-EMS systems can be successfully implemented globally, especially in areas with limited healthcare infrastructure. As new diagnostic technologies, such as portable ultrasound devices, become more widely available for use by paramedics, further research could assess the impact of these tools on the diagnostic accuracy of tele-EMS physicians. Studies could explore whether tele-EMS support combined with real-time diagnostic data can further improve prehospital diagnoses, particularly in complex cases like dyspnea, abdominal pain, or trauma.

## Data Availability

The raw data supporting the conclusions of this article will be made available by the authors, without undue reservation.

## TEMS-study group collaborators

Julian Bartman, Lars Becker, Stefan Beckers, Ladin Bozlu, Jörg Brokmann, Mark Coburn, Michael Czaplik, Gholamreza Fazlipour, Marc Felzen, Christina Fitzner, Andreas Follmann, Linda Grüßer, Alexander Gunesch, Patrick Hess, Ralf-Dieter Hilgers, Michael Holten, Thea Koch, Ana Kowark, Peter Kranke, Julia Liebens, Michael Reugels, Ute Roschanski, Rolf Rossaint, Jane Schroeder, Hanna Schröder, Pia Stadler, Carla Tutlies, Stephanie Wied, Sebastian Ziemann. We would like to express our gratitude to the employees of the EMS in the city of Aachen for their hard work every day. We also want to thank alle contributors of the TEMS study, particularly for their efforts in the complex data collection process.
